# Reduction of Peripheral Blood iNKT and γδT Cells in Patients With Parkinson's Disease: An Observational Study

**DOI:** 10.3389/fimmu.2020.01329

**Published:** 2020-06-25

**Authors:** Chao Zhou, Xinhua Zhou, Dan He, Zhen Li, Xufang Xie, Yue Ren

**Affiliations:** ^1^Department of Neurology, Jiangxi Provincial People's Hospital Affiliated to Nanchang University, Nanchang, China; ^2^The Neurological Institute of Jiangxi Province, Jiangxi Provincial People's Hospital Affiliated to Nanchang University, Nanchang, China; ^3^Department of Neurology, The First Affiliated Hospital of Nanchang University, Nanchang, China

**Keywords:** Parkinson's disease, iNKT cells, γδT cells, neuroinflammation, T cell subset

## Abstract

**Objective:** To investigate the frequencies and numbers of invariant natural killer T (iNKT) cells and γδT cells in the peripheral blood of patients with the Parkinson's disease (PD), and to examine their association with the PD severity.

**Methods:** Peripheral blood samples from 47 PD patients (PD group) and 47 age-matched healthy control subjects (HC group) were collected. The frequencies and the absolute cell numbers were analyzed by flow cytometry. Mann-Whitney *U*-test was used to test the difference between two groups, where *P* < 0.05 was considered as significant. An ordered probit regression method was used to examine the association of the iNKT and γδT cells with severity of PD.

**Results:** Patients in the PD group showed significantly lower frequencies (0.039 vs. 0.139%; *P* = 0) and cell counts (308/mL vs. 1,371/mL; *P* = 0) of iNKT cells compared to the HC group. Moreover, the percentages and absolute numbers of γδT cells were significantly decreased in the PD group compared to the HC group (3.69 vs. 7.95% and 30/μL vs. 66/μL; *P* = 0). The iNKT cells were significantly reduced in PD patients with higher Unified Parkinson's Disease Rating Scale (UPDRS) scores or cognitive decline.

**Conclusions:** Cell frequencies and absolute numbers of iNKT cells and γδT cells are significantly reduced in the peripheral blood samples of PD patients. Patients with high UPDRS scores or cognitive decline also showed significant reduction of iNKT cells. Our results suggest that iNKT cells and γδT cells may contribute to the development of PD.

## Introduction

Parkinson's disease (PD) is a neurodegenerative disorder that commonly affects individuals of >40 years old. An early and progressive loss of the dopaminergic neurons in the substantia nigra and abnormal aggregation of α-synuclein are believed to contribute to the development of the disease (1). Currently, there is no standard objective diagnostic criteria for the PD and the diagnostic approach of this disease mainly relies on its clinical manifestations, including resting tremors, rigidity, and bradykinesia (1–3). Since an early diagnosis of PD is particularly difficult due to non-specific or barely noticeable symptoms that are present at early stages of the disease, the main focus of the PD research field addresses issues associated with its prevention, early diagnosis, and treatment approaches.

Although the etiology of PD is not fully elucidated, neuroinflammation is believed to be one of the mechanisms contributing to the PD pathology. It is well-known that neuroinflammation plays an important role in the pathogenesis of multiple neurodegenerative diseases, including Alzheimer's disease (AD), amyotrophic lateral sclerosis (ALS), and PD. Although the neuro-immune effects and processes differ in each of above-mentioned pathologies, the altered innate immune responses, followed by an unbalance of pro- and anti-inflammatory cytokines, are commonly shared features within these multiple neurodegenerative diseases. In line with this proposed mechanism, several studies demonstrated the activation of brain microglia in PD patients (4). A recent study has reported the antigenic epitopes of the α-synuclein in PD patients can be recognized by helper and cytotoxic T cells (5). Furthermore, these activated T cells were demonstrated to trigger the autoimmune reactions attacking the dopamine neurons, which results in the death of these neurons (5). Altogether above studies suggested that PD might be highly associated with the activation status of immune cells in the central neural system (CNS). This association is also evidenced by changes in the composition of immune cell subsets, such as decreased proportion of CD4^+^ T cells with increased ratio of Th1/Th2 cells as well as increase in Th17 cells and myeloid-derived suppressor cells (MDSC) in the peripheral blood (6, 7). Moreover, the proinflammatory cytokines, including TNF-α, IL-1β, IL-2, IL-6, and INF-γ, were up-regulated in the brain tissues, cerebrospinal fluid (CSF) and serum of PD patients (8), that collectively represent characteristic system-wide or CNS-specific chronic inflammatory changes often seen in PD patients.

Vα24Jα18 (in humans) or Vα14Jα18 (in mice) T cell receptor (TCR) α-chain expressing invariant natural killer T (iNKT) cells comprise a unique T cell subset that expresses surface markers characteristic of both T cells and natural killer (NK) cells, whereas γδT cells are a T cell subset that expresses γδ TCR. The iNKT cells can be divided into several subpopulations based on their cytokine profiles, such as the iNKT1, iNKT2, and iNKT17, which produce the Th1, Th2, and Th17 cytokines, respectively (9, 10). Similarly, γδT cells can be subdivided based on their cytokine profiles similar to those of iNKT cells (11). Unlike helper T cells, both iNKT and γδT cells represent functionally mature cells that can rapidly secrete large amounts of cytokines in response to various stimuli without a need for the prior sensitization to undergo functional maturation culminating in a clonal expansion of effector cells that is the hallmark feature of the adaptive immune response. Thus, iNKT and γδT cells are thought to act as immune regulators connecting the innate and adaptive immune systems, and to mediate various immune responses by virtue of their potential to produce massive amounts of a wide range of cytokines and chemokines upon activation. Albeit intense studies have enormously advanced our knowledge on the biology of these unique cell types, it still remains unascertained regarding the involvement of iNKT and γδT cells in the course of neuroinflammatory conditions such as the PD.

In order to investigate the possible involvement of iNKT and γδT cells in the pathogenesis of PD, we examined in this study the frequencies and cell counts of iNKT and γδT cells in the peripheral blood of PD patients, and analyzed their association with the disease stage and severity. Our results demonstrate that the cell frequencies and numbers of peripheral blood iNKT cells and γδT cells are significantly reduced in PD patients. Our data also imply that PD patients with higher UPDRS scores or cognitive decline possess significantly reduced peripheral blood iNKT cells. We hope our findings will stimulate more in-depth research in this field.

## Materials and Methods

### Study Subjects

A total of 47 PD patients were recruited from the Jiangxi Provincial People's Hospital from August 2017 to October 2019. The diagnosis of PD was done according to the United Kingdom Parkinson's Disease Brain Bank criteria. None of the patients had a previous treatment at the time of admission. The severity of the PD was evaluated according to the Unified Parkinson's Disease Rating Scale scores (UPDRS) (12) and the Hoehn and Yahr (H&Y) scale (13). A total of 47 individuals in the control group were recruited from the healthy subjects who underwent a routine health checkup procedure during the same time period at the aforesaid hospital. Both groups were selected based on the exclusion criteria: (1) age ≥ 80; (2) pregnant or breast-feeding; (3) severe heart and lung dysfunction; (4) stroke; (5) Alzheimer's disease; (6) cerebrovascular deformation; (7) infection; (8) cancer; (9) hematology diseases; (10) immune compromised diseases; (11) diseases of the connective tissues; (12) trauma or surgery in the recent 3 months; (13) undergoing anti-inflammatory or immune-inhibitory treatment.

### Flow Cytometry

Two milliliters of peripheral blood were collected in EDTA-tube. After collection, blood samples were incubated with fluorescence labeled monoclonal antibodies to define immune cell subpopulations. Antibodies (BD Biosciences or BioLegend) used for flow cytometry were: FITC-CD3 and APC-TCR Vα24-Jα18 or FITC-CD3 and PE-TCRγ/δ to stain iNKT cells or γδT cells, respectively. Cells were analyzed by Mindray BriCyte E6 (Mindray). The percentage and cell count of iNKT cells (CD3^+^, TCR Vα24-Jα18^+^) and γδT cells (CD3^+^, TCR γ/δ^+^) were determined using FlowJo software (BD Biosciences).

### Statistical Analyses

Descriptive statistical analyses were performed using SPSS 19.0. Data were presented as Mean ± SD, or Median (IQR). The Mann-Whitney *U*-test in SPSS was used to test statistical differences between two groups, where *P* < 0.05 was considered significant. The ordered probit regression adjusting for subjects' age and sex was performed to test the association of iNKT and γδT cells with the PD stage measured by H&Y scale, and mixed model adjusting for age, sex, duration of disease, motor complications, gait-disturbances, and cognitive decline, was used to test the associations with PD severity measured by UPDRS and UPDRS III. Analyses were performed using Stata (IC 16.0). The H&Y stage and UPDRS score of healthy controls were set to 0. As the cell count and percentage were highly correlated, we have performed the regression separately for the cell count and percentage.

## Results

### Characteristics of Study Participants

In the present study, a total of 47 (29 male and 18 female) PD patients (PD group) were recruited. The age of PD patients ranged from 37 to 76, with an average age of 61.85 ± 8.89 years. Number of healthy control (HC group) subjects was 47, including 22 males and 25 females. The age of healthy controls ranged from 44 to 72 years with an average age of 58.83 ± 7.38 years. The mean disease duration was 4.38 ± 4.35 years in PD patients, with an average age of onset at 57.60 ± 9.68 years old. The average of H&Y stage and UPDRS scores of PD group were 2.43 ± 1.00 and 54.15 ± 38.16, respectively, and that of the UPDRS III was 20.36 ± 13.14. Within 47 PD patients, 13 presented motor complications, 30 presented gait disturbances and 9 presented cognitive decline (Table 1).

**Table 1:** Characteristics and clinical indices of study participants.

**Demographics**	**PD (*n* = 47), HC (*n* = 47)**
Age (years)	61.85 ± 8.89, 58.83 ± 7.38
Gender (M/F)	29/18, 22/25
**Clinical characteristics of PD patients**	
Duration of disease (years)	4.38 ± 4.35
Age of onset	57.60 ± 9.68
H&Y stage	2.43 ± 1.00
UPDRS	54.15 ± 38.16
UPDRS III	20.36 ± 13.14
Motor complications	13 (34)
Gait disturbances	30 (17)
Cognitive decline	9 (38)

*Data were presented as Mean ± SD. PD, Parkinson's disease group; HC, healthy control group; M, Male; F, Female; H&Y stage, Hoehn–Yahr stage; UPDRS, Unified Parkinson's Disease Rating Scale; UPDRS III, motor function evaluation*.

### Peripheral Blood iNKT Cells Are Decreased in Patients With PD

**Figure 1:**
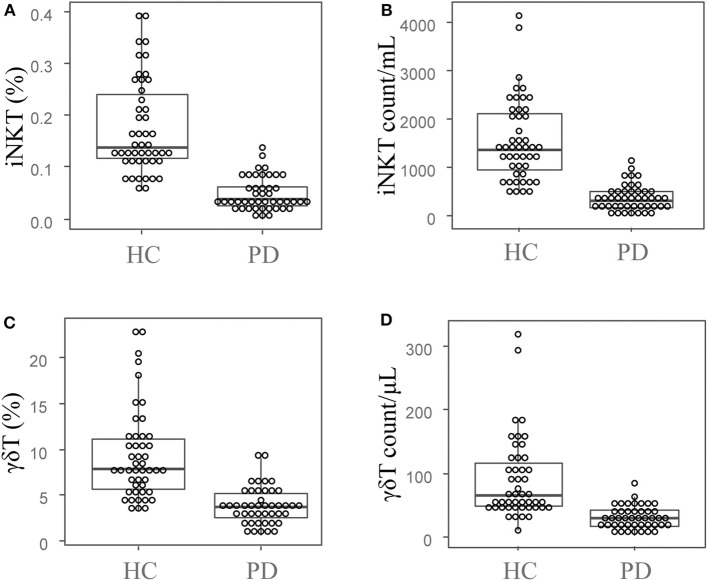
Peripheral blood iNKT cells and γδT cells are decreased in PD patients. The dot plots and boxplots of the peripheral blood iNKT cell percentages **(A)** and cell counts **(B)**, as well as the peripheral blood γδT cells percentages **(C)** and cell counts **(D)**. PD, Parkinson's disease group; HC, health control group.

Frequencies and absolute cell numbers of peripheral blood iNKT cells from PD group and HC group were analyzed by flow cytometry. As shown in Figure 1A, the median (IQR) percentage of iNKT cells in PD group was 0.039 (0.05)%, which was significantly lower than that in HC group [0.139 (0.13)%, *U* = 110, *Z* = −7.402, *P* = 0]. Moreover, the median iNKT cell count of PD group [308 (322)/mL] was also significantly decreased as compared with HC group [1,371 (1,225)/mL] by the Mann-Whitney *U*-test (*U* = 85, *Z* = −7.596, *P* = 0; Figure 1B). Our results indicate a significant reduction of the peripheral blood iNKT cells in PD patients as compared with healthy controls.

### Peripheral Blood γδT Cells Are Decreased in PD Patients

We also investigated the frequencies and absolute cell numbers of peripheral blood γδT cells of PD and HC groups by flow cytometry. The median (IQR) percentage of γδT cells in PD group was 3.69 (2.98)%, and 7.95 (5.47)% in HC group. The Mann-Whitney U test showed significant difference between PD and HC groups (*U* = 232, *Z* = −6.37, *P* = 0; Figure 1C). Additionally, the median (IQR) cell count of γδT cells in PD group [30 (27)/μL] was also significantly lower than that of in HC group [66 (76)/μL; *U* = 244, *Z* = −6.275, *P* = 0; Figure 1D]. These results indicate that the peripheral blood γδT cells are significantly decreased in PD patients compared with healthy controls.

### Association of iNKT and γδT Cells With the PD Severity

Next we examined whether iNKT and γδT cells are associated with the PD severity by using an ordered probit regression model by controlling the age and sex. With the adjustment of the age and sex, both iNKT and γδT cells were negatively associated with the H&Y stage, UPDRS and UPDRS III scores when healthy controls were included in the data modeling (*P* = 0; Table 2, Supplementary Figures 1–3). However, when we performed the same analysis by excluding healthy controls from the analysis and additionally adjusted for the age of onset, duration of disease, and whether disease complications of PD were present, the percentage and cell counts of iNKT cells showed marginal significant negative correlation with the H&Y stage (*P* = 0.071 and 0.084, respectively; Table 2, Supplementary Figure 1). However, no significant associations were observed for iNKT and γδT cells with the disease severity estimated by UPDRS and UPDRSIII (Table 2, Supplementary Figures 2, 3).

**Table 2:** The estimated coefficients of the ordered probit regression models of H&Y stage, UPDRS, and UPDRSIII scores in the whole dataset and the PD group only.

**Samples**		**H&Y**			**UPDRS**			**UPDRS III**		
		**Coefficient**	**SE**	***P*-value**	**Coefficient**	**SE**	***P*-value**	**Coefficient**	**SE**	***P*-value**
PD and HC	iNKT%	−24.162	3.874	0.000	−188.277	35.651	0.000	−70.162	13.047	0.000
	γδT%	−0.300	0.057	0.000	−3.306	0.745	0.000	−1.225	0.274	0.000
	iNKT#	−0.003	0.000	0.000	−0.021	0.004	0.000	−0.008	0.001	0.000
	γδT#	−0.034	0.007	0.000	−0.254	0.065	0.000	−0.097	0.024	0.000
PD only	iNKT%	−11.139	6.159	0.071	−60.686	108.256	0.575	−17.961	48.396	0.711
	γδT%	0.039	0.096	0.681	0.768	1.748	0.660	0.272	0.786	0.729
	iNKT#	−0.001	0.001	0.084	−0.012	0.014	0.383	−0.008	0.006	0.191
	γδT#	−0.001	0.011	0.904	−0.120	0.199	0.547	−0.124	0.088	0.159

*%, percentage; #, cell count; PD, Parkinson's disease group; HC, health control group; H&Y, Hoehn–Yahr stage; UPDRS, Unified Parkinson's Disease Rating Scale; UPDRS III, motor function evaluation; SE, standard error; CI, confidence interval*.

**Figure 2:**
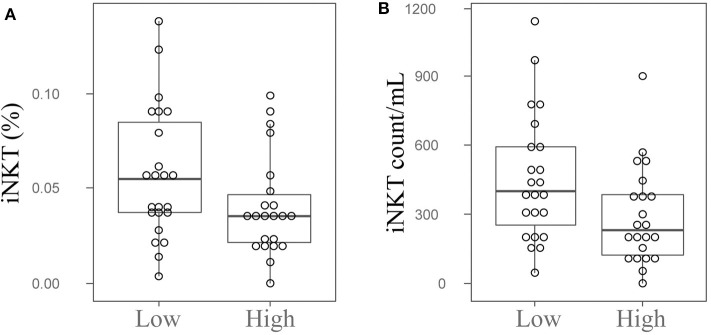
Peripheral blood iNKT cells are decreased in the PD patients with high UPDRS scores. The dot plots and boxplots of the peripheral blood iNKT cells percentages **(A)** and cell counts **(B)** in PD patients with high or low UPDRS scores. Low, PD patients with UPDRS score <49; High, PD patients with UPDRS score ≥49; UPDRS, Unified Parkinson's Disease Rating Scale.

Since we observed that patients with higher UPDRS scores possess lower numbers of iNKT cells (Supplementary Figure 2), we divided PD patients into two groups by the median value of UPDRS score (median = 49), and then compared the numbers and percentages of iNKT cells between two groups. As shown in Figure 2, both frequencies and absolute numbers of iNKT cells were significantly lower in patients with higher UPDRS scores than those with lower UPDRS scores (*P* = 0.049 and 0.033 for cell frequency and cell count). Interestingly, when PD patients were grouped according to their presence of PD symptoms such as motor complications, gait disturbance and cognitive declines, a significant reduction of iNKT cells was observed in patients with the cognitive decline (*P* = 0.007 and 0.002 for cell percentage and cell count) (Supplementary Figures 4A,B). These results suggest that the reduced numbers of iNKT cells in PD patients might be associated with the PD severity.

## Discussion

It is well-accepted that the neuroinflammation plays an important role in the pathogenesis of the PD (14). Several studies have demonstrated alterations in the composition of immune cell subpopulations and levels of cytokines detected in the peripheral blood and CSF specimens of PD patients. Decreased numbers of T lymphocytes with the significantly decreased frequency of CD4^+^ T cells were reported to be present in the peripheral blood of PD patients. Also, it was reported that CD4^+^ and CD8^+^ T cells were detected at significantly higher numbers in the substantia nigra of PD patients compared with those of healthy controls (15). Furthermore, PD patients were shown to possess a shift toward the Th1 type immune response with increased levels of IFN-γ, and reduced number and suppressive capacity of Treg cells, reduced number of B lymphocytes as well as increased number of NK cells (6, 16–19). Additionally, Schröder et al., reported about a shift in the composition of monocyte subsets and an activation of T lymphocytes in the CSF of PD patients (20), which provided further evidence for the activated statuses of both innate and adaptive immune systems observed in the disease course of the PD. iNKT and γδT cells have important roles in the immune regulation by bridging the innate and acquired immune responses. However, it was still unexplored regarding the roles of iNKT and γδT cells in the PD. Thus, in order to address this question, we have analyzed the peripheral blood samples obtained from the PD patients. The results shown in the present study demonstrate a significant reduction of the peripheral iNKT and γδT cells in the PD patients, which suggests strongly for a possible involvement of these cell types in the PD pathogenesis.

Moreover, it has been reported that peripheral blood CD4^+^ T cells are negatively correlated with the H&Y stage and UPDRS score, and B cells are negatively correlated with the H&Y stage in PD patients (19). Studies have also indicated that the activation of effector memory T cells and the dysfunction of regulatory T cells may be linked to the PD pathobiology, disease severity, and especially to the motor dysfunction (6). Moreover, increase in NK cells and decrease in Th1 cells in the peripheral blood of PD patients were reported to be positively correlated with UPDRS scores (18). In our present study, both iNKT and γδT cells were negatively associated with the H&Y stage, UPDRS, and UPDRS III scores when PD and HC subjects were all taken into analysis. However, we were not able to identify significant associations of iNKT and γδT cells with the PD severity estimated by UPDRS scores within PD patients. Nonetheless, it was found that the high score group showed significant reduction of iNKT cells upon segregation of PD patients into groups with low and high UPDRS scores. Moreover, iNKT cells were also significantly reduced in PD patients with the cognitive decline. Thus, our results suggest that these cell types might be critical for the disease development, where iNKT cells might be especially associated with the disease progression.

Although studies in the PD are very limited, investigations on iNKT and γδT cells in the MS, a well-known immune-mediated CNS demyelinating disease, may provide insights into their roles in neuroinflammatory responses observed in the PD. Previous studies have reported that the MS patients show a significant reduction in iNKT cells with an altered cytokine secretion profiles but an accumulation of γδT cells in the CSF and pathologic tissue specimens (21, 22), suggesting iNKT and γδT cells may play some intricate roles on the development of neuroinflammatory responses. It is well-known that iNKT and γδT cells, which can be further divided into subgroups based on their cytokine producing characteristics, possess a unique potential to directly regulate immune responses through their rapid and massive production of a wide range of cytokines, or indirectly through their regulation of other immune cell types (10, 11). A study found that iNKT cells produce a large amount of Th2 cytokines indicative of a significant bias toward Th2 type response during the MS remission phase or after the treatment (22). Additionally, in the experimental autoimmune encephalomyelitis (EAE, a mouse model for MS disease), activated iNKT cells produced increased levels of IL-4 and promoted the CNS infiltration with proinflammatory monocytes that differentiate toward the anti-inflammatory M2 macrophages alleviating the symptoms of EAE (23). Interestingly, it was reported that the CNS-infiltrating CD3^+^ T lymphocytes can regulate the transition of microglia from the anti-inflammatory M2 to the proinflammatory M1 phenotype by using the Thy1-WTS transgenic mice, which are known as a mouse model for PD (24). One of possible roles of iNKT cells in the PD pathogenesis could be a scenario, where iNKT cells promote the differentiation of CNS-infiltrating macrophages toward M2-type phenotype that could possibly result in the M1/M2 imbalance skewed toward the proinflammatory phenotype. In the EAE model, γδT cell is one of the major sources of IL-17 (25). A previously published study found that γδT cells can inhibit the function of Treg cells to promote the autoimmune process (26). While some noticeable alterations of the Treg cell function and frequency were observed in PD patients (6), whether this is related to the reduction of peripheral blood γδT cells is yet to be investigated.

It is important to mention about our limitations which might render the results of this study not to be representative of the whole population mainly due to a small sample size. Nonetheless, our results point out also for the need of further investigations with larger cohort size with specimens collected from possibly many different hospitals that would ideally result in validation of our results using the meta-analysis with a better statistical power. Although our study does not provide mechanistic insights into the roles of iNKT and γδT cells in the development of the PD, we believe that our findings on the reduction of iNKT and γδT cells in PD patients will definitely stimulate a research in this field.

In summary, this is the first study to report the reduction of peripheral blood iNKT and γδT cells in PD patients. The reduction of iNKT cells seems to be significantly associated with the PD disease progress stages. Our study suggests a possible role of iNKT and γδT cells in the PD pathogenesis and progression. We believe our findings warrant further in-depth research in the PD field that will hopefully lead to better understanding of the disease and help in designing early diagnostic methods as well as preventive strategies of the PD.

## Data Availability Statement

The datasets generated for this study are available on request to the corresponding author.

## Ethics Statement

The studies involving human participants were reviewed and approved by the Institution Review Board (IRB) at Jiangxi Provincial People's Hospital Affiliated to Nanchang University, Nanchang, China. The patients/participants provided their written informed consent to participate in this study.

## Author Contributions

CZ contributed to recruitment of patients, data acquisition and analysis, and manuscript writing. XZ examined patients and collected clinical data. DH and ZL performed FACS analyses. XX contributed to the study design and to the supervision of the clinical part of the study, and made critical revision of the manuscript. YR contributed to the study design, supervision of the study, interpretation of data, and wrote the manuscript. All authors contributed to the article and approved the submitted version.

## Conflict of Interest

The authors declare that the research was conducted in the absence of any commercial or financial relationships that could be construed as a potential conflict of interest.
