# Supplementary material for: Reduction of Peripheral Blood iNKT and γδT Cells in Patients With Parkinson's Disease: An Observational Study
Source: Front Immunol. 2020 Jun 25;11:1329. doi: 10.3389/fimmu.2020.01329 (PMC7330172; doi:10.3389/fimmu.2020.01329)
Supplement: Supplementary file 1 [file Image_1.PDF]

Supplementary Material

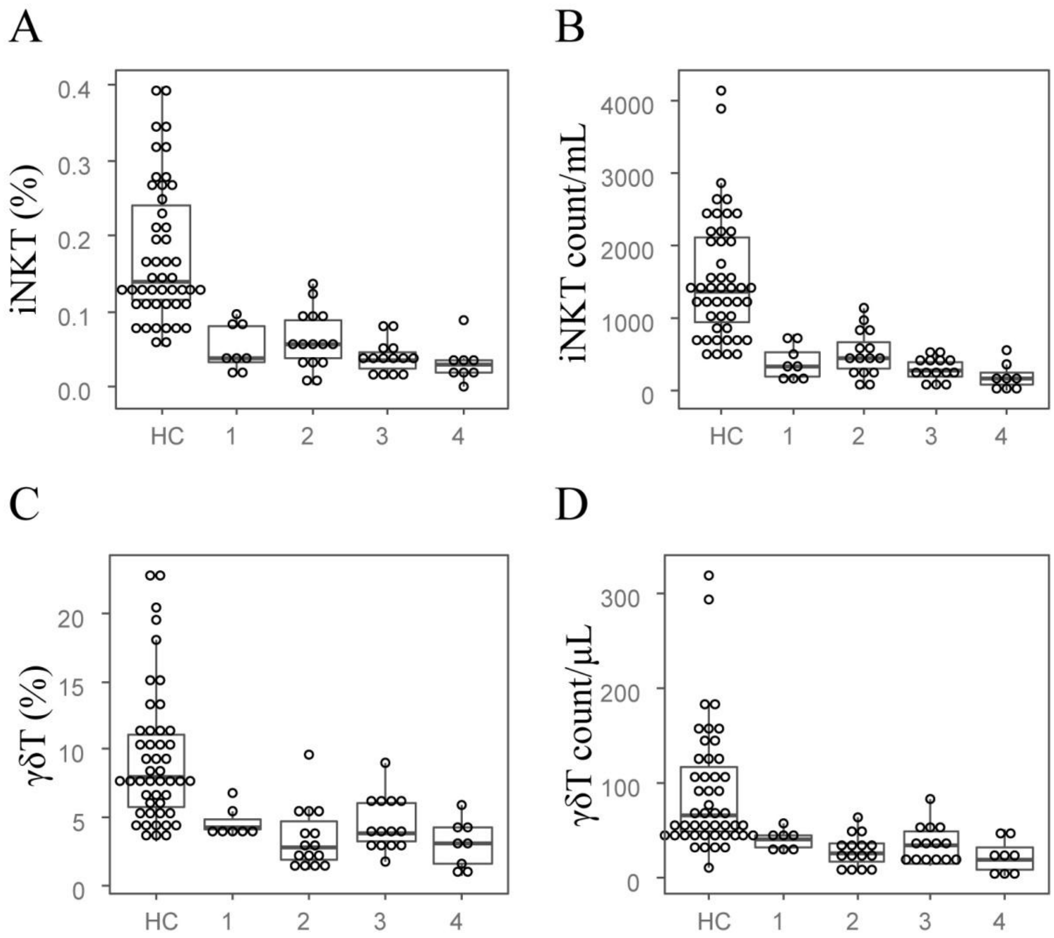

Supplementary Figure 1. The dot plots and boxplots of the peripheral blood iNKT cell percentages (A), iNKT cell counts (B), γδT cell percentages (C), and γδT cell counts (D) by the H&Y stage of the HC group (H&Y stage=0) and the PD group (H&Y stage>0). HC, health control group.

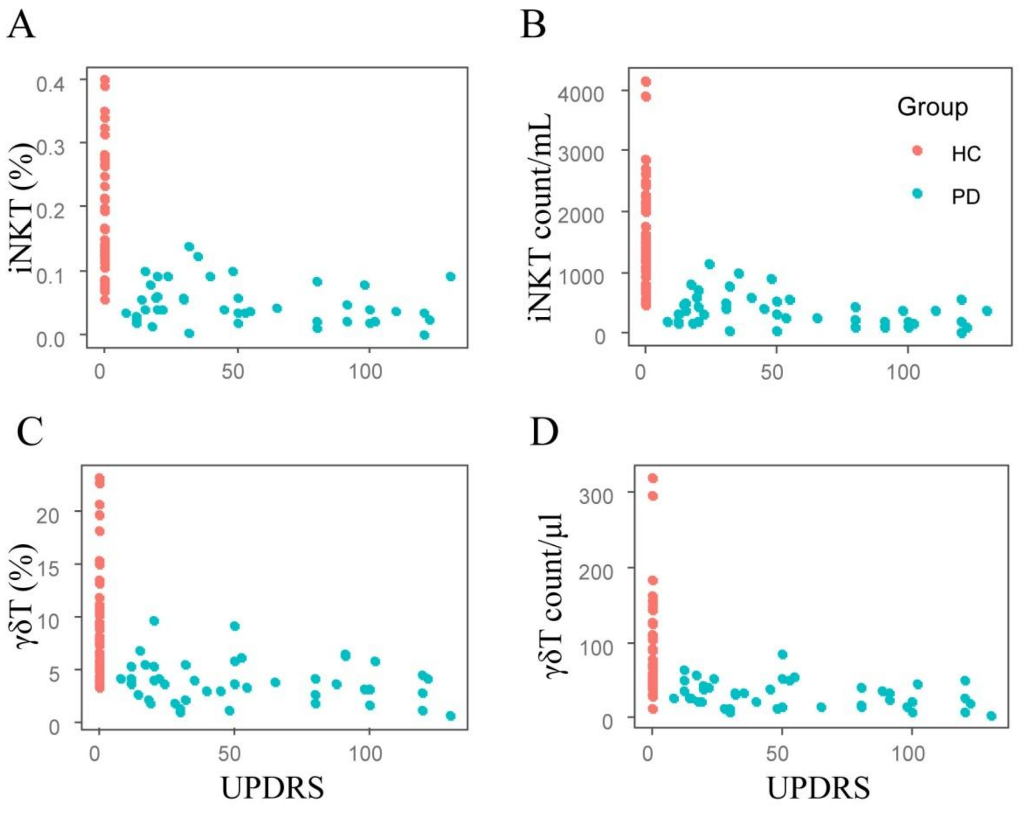

Supplementary Figure 2. The scatter plots of the peripheral iNKT cell percentage (A), iNKT cell count (B), γδT cell percentage (C), and γδT cell count (D) by UPDRS of the HC group (UPDRS=0, pink dots) and the PD group (UPDRS>0, cyan dots).

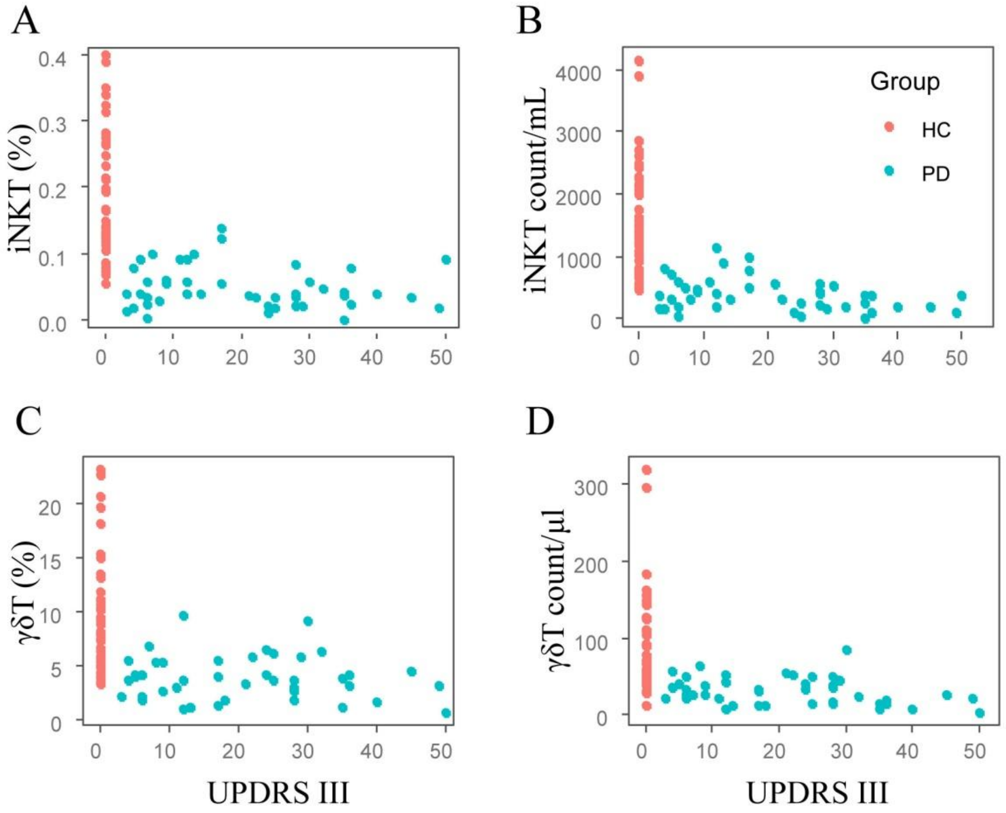

Supplementary Figure 3. The scatter plots of the peripheral iNKT cell percentage (A), iNKT cell count (B), γδT cell percentage (C), and γδT cell count (D) by UPDRS III of the HC group (UPDRS III=0, pink dots) and the PD group (UPDRS III >0, cyan dots).

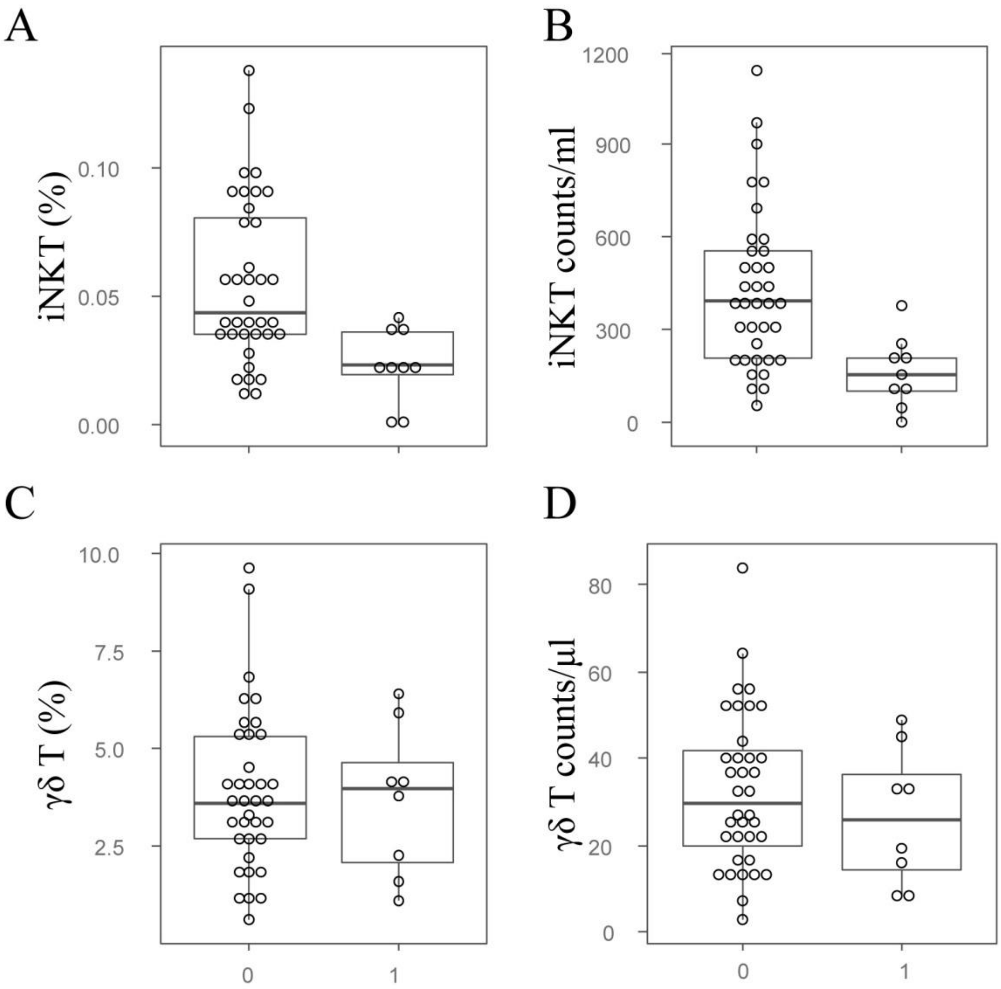

Supplementary Figure 4. The dot plots and boxplots of the peripheral blood iNKT cell percentages (A) and cell counts (B), as well as the peripheral blood γδT cells percentages (C) and cell counts (D) in PD patients with cognitive decline or not. 0, PD patients without cognitive decline; 1, PD patients with cognitive decline.
